# Impact of antimicrobial use on abundance of antimicrobial resistance genes in chicken flocks in Vietnam

**DOI:** 10.1093/jacamr/dlad090

**Published:** 2023-07-22

**Authors:** Nguyen Thi Nhung, Avijit Dutta, Ellen Higginson, Leanne Kermack, Nguyen Thi Phuong Yen, Doan Hoang Phu, Bach Tuan Kiet, Marc Choisy, Ronald B Geskus, Stephen Baker, Juan Carrique-Mas

**Affiliations:** Oxford University Clinical Research Unit, Ho Chi Minh City, Vietnam; Cambridge Institute of Therapeutic Immunology & Infectious Disease, University of Cambridge, Cambridge, UK; Chattogram Veterinary and Animal Sciences University, Chattogram 4225, Bangladesh; Cambridge Institute of Therapeutic Immunology & Infectious Disease, University of Cambridge, Cambridge, UK; Cambridge Institute of Therapeutic Immunology & Infectious Disease, University of Cambridge, Cambridge, UK; Oxford University Clinical Research Unit, Ho Chi Minh City, Vietnam; Oxford University Clinical Research Unit, Ho Chi Minh City, Vietnam; Faculty of Animal Science and Veterinary Medicine, Nong Lam University, Ho Chi Minh City, Vietnam; Sub-Department of Animal Health and Production, Dong Thap Province, Cao Lanh, Vietnam; Oxford University Clinical Research Unit, Ho Chi Minh City, Vietnam; Centre for Tropical Medicine and Global Health, University of Oxford, Oxford, UK; Oxford University Clinical Research Unit, Ho Chi Minh City, Vietnam; Centre for Tropical Medicine and Global Health, University of Oxford, Oxford, UK; Cambridge Institute of Therapeutic Immunology & Infectious Disease, University of Cambridge, Cambridge, UK; Oxford University Clinical Research Unit, Ho Chi Minh City, Vietnam; Centre for Tropical Medicine and Global Health, University of Oxford, Oxford, UK; Food and Agriculture Organization of the United Nations (FAO), Hanoi, Vietnam

## Abstract

**Objectives:**

We investigated longitudinally Vietnamese small-scale chicken flocks in order to characterize changes in antimicrobial resistance gene (ARG) content over their life cycle, and the impact of antimicrobial use (AMU) on an intervention consisting of veterinary advice provision.

**Methods:**

AMU data and faecal samples were collected from 83 flocks (25 farms) at day-old, mid- and late-production (∼4 month cycle). Using high-throughput real-time PCR, samples were investigated for 94 ARGs. ARG copies were related to 16S rRNA and ng of DNA (ngDNA). Impact of AMU and ARGs in day-olds was investigated by mixed-effects models.

**Results:**

Flocks received a mean (standard error, SE) animal daily dose (ADD) of 736.7 (83.0) and 52.1 (9.9) kg in early and late production, respectively. Overall, ARGs/16S rRNA increased from day-old (mean 1.47; SE 0.10) to mid-production (1.61; SE 0.16), further decreasing in end-production (1.60; SE 0.1) (all *P* > 0.05). In mid-production, ARGs/16S rRNA increased for aminoglycosides, phenicols, sulphonamides and tetracyclines, decreasing for polymyxins β-lactams and genes that confer resistance to mutiple classes (multi-drug resistance) (MDR). At end-production, aminoglycoside resistance decreased and polymyxin and quinolone resistance increased (all *P* < 0.05). Results in relation to ngDNA gave contradictory results. Neither AMU nor ARGs in day-olds had an impact on subsequent ARG abundance. The intervention resulted in 74.2% AMU reduction; its impact on ARGs depended on whether ARGs/ngDNA (+14.8%) or ARGs/16S rRNA metrics (−10.7%) (*P* > 0.05) were computed.

**Conclusions:**

The flocks’ environment (contaminated water, feed and residual contamination) is likely to play a more important role in transmission of ARGs to flocks than previously thought. Results highlight intriguing differences in the quantification of ARGs depending on the metric chosen.

## Introduction

Antimicrobial-resistant (AMR) bacterial infections are estimated to cause hundreds of thousands of deaths globally, resulting in a significant economic burden.^1^ Excessive use of antimicrobials in human communities and hospitals, as well as in animal production, is a major driver for the emergence and dissemination of AMR. Notably, approximately 75% of antimicrobial use (AMU) globally is associated with animal production.^2,3^

Poultry meat (predominantly chicken) is the most consumed protein commodity worldwide. Due to fast population growth and rising incomes the global consumption of poultry meat has been rising and is expected to reach 150 million tonnes by 2029.^4^ In Vietnam, chicken consumption has increased 119% (from 451 000 to 990 000 tonnes) between 2010 and 2020, ranking as the second most commonly consumed type of meat after pork.^5,6^

Globally, chickens are dosed with antimicrobials more than any other species [mean 138 animal daily doses (ADDs) per 1000 chicken-days].^7^ In connection with the increasing demand for meat, antimicrobial consumption in chicken production in Asia is predicted to raise by 143% from 2010 to 2030.^8^ In Vietnam and other low- and middle-income countries (LMICs), farming of small-scale chicken flocks is particularly common. Levels of AMU in such farms often reflect a situation of high incidence of disease in flocks,^9^ as well as easy access to antimicrobials over the counter.^10^ A study in the Mekong Delta (Vietnam) showed that a total of 383 ADDs of antimicrobials per 1000 chicken-days were typically administered to small-scale flocks, mostly through the water route.^11^ One of the primary public health concerns is the over-reliance on critically important antimicrobials (CIAs) in chicken farming.^11,12^

Despite the importance of AMR in poultry farming, very few studies have investigated changes in AMR over the flock production cycle.^13–16^ Studies in the Mekong Delta region have documented an increase in colistin and gentamicin resistance in *Escherichia coli* from two-month-old chickens compared with day-old chicks.^14,15^ In contrast, decreases in prevalence of resistance to tetracyclines and sulphonamides have been reported in *E. coli* isolated from day-old chicks to maturity in laying hens in Europe.^16^ Using longitudinal data from chicken flocks in the Mekong Delta (Vietnam), a modelling study on colistin resistance indicated that AMU in the early stage of production or introduction from hatcheries is far less determinant than importation from the environment and use in latter stages.^17^

Associations between AMU and phenotypic resistance in animal production have been shown using country-level data across the EU.^18^ Also, studies on broiler flocks in China and Europe have reported a correlation between abundance of antimicrobial resistance genes (ARGs) and antimicrobial exposure.^19,20^ In Vietnam, studies on chicken flocks have reported an impact of AMU on phenotypic (colistin, ciprofloxacin) and genotypic resistance (i.e. ARGs such as *mcr-1*, *gyrA* and *strAB*).^15,21^ Establishing the precise relationship between AMU and AMR requires an intense sampling schedule matched with high-quality AMU data, both of which are difficult to obtain in many small-scale farming settings.

An intervention study targeting small-scale native chicken flocks consisting of regular provision of veterinary advice to farmers resulted in a 66% reduction in AMU.^22^ To our knowledge, no studies have investigated the impact of AMU on ARG content in a high-AMU hotspot such as the Mekong Delta of Vietnam. Using a high-throughput real-time quantitative PCR (HT-qPCR) platform for a comprehensive ARG panel coupled with detailed AMU data, we aimed to investigate the dynamics of ARGs over the chicken flock production cycle as well as the impact of oral administration of antimicrobials.

## Methods

### Ethics

Written informed consent was obtained from all farm owners on their participation in the study. The study was granted ethics approval by the Oxford University Ethics Committee (OxTREC, Ref. No. 5121-16).

### Farms and flocks

We investigated 83 small-scale flocks from 25 farms that had been previously recruited to an intervention trial in two districts in Dong Thap province in the Mekong Delta of Vietnam. The study design and the criteria for farm selection have been previously described.^22,23^ The study included a baseline phase (October 2016 to July 2018) followed by an intervention phase, which consisted of the provision of farmer training and on-farm veterinary advice (April 2018 to November 2019).

### Sample and AMU data collection

From each flock, pooled faecal samples and AMU data were collected at three timepoints along the production cycle: (i) day-old chicks (i.e. on arrival to the farm from the hatchery); (ii) mid-production (∼8 weeks); and (iii) end-production (i.e. immediately before depopulation) (∼16 weeks) by staff affiliated to the Dong Thap Sub-Department of Animal Health and Production (SDAH-DT). Mid- and end-production faecal material was collected by placing 3–5 paper liners (50 × 50 cm) around feeders and drinking areas. The liners were collected after 60–90 min or when at least 10 faecal droppings were visible. A hand-held sterile gauze was used to swab visible faecal contents on the liners. In the case of day-olds, the crates were swabbed directly. Each swab was placed in a universal jar and mixed vigorously with 50 mL of saline buffer. The resulting eluate (850 μL) was mixed with glycerol (150 μL) and stored at −20°C. Weekly data on AMU and farming practices were collected as described previously.^22^

### ARG panel

The primer set for the qPCR assays comprised 94 ARGs belonging to 13 different antimicrobial classes (Table S1, available as Supplementary data at *JAC-AMR* Online). Primers were designed for ARGs that confer resistance against the most commonly used antimicrobials in chicken farms in the area,^11^ as well as clinically relevant ARGs, including those encoding ESBLs and vancomycin resistance. A 16S rRNA marker was also included to assess bacterial biomass.

### Laboratory processing of samples

DNA from pooled faecal samples (∼0.1 g per sample) was extracted using the QIAamp DNA Stool Mini kit (QIAGEN, Germany) and was quantified using a Nanodrop 2000 spectrophotometer (Thermo Fisher, USA). DNA was diluted to a working concentration of 10 ng/µL. HT-qPCR for detection of ARGs was performed using the 96.96 Biomark Dynamic Array™ for Real-time PCR (Standard BioTools, USA). First, faecal DNA (1.25 µL) was subjected to 12 cycles of specific target amplification using 3.75 µL of pre-mix (Preamp Master Mix, mixture of all primer set and nuclease-free water). The amplified sample was then cleaned up using exonuclease I followed by a 5-fold dilution prior to loading onto the 96.96 integrated fluidic circuit (IFC). Final thermal cycling along with real-time imaging was performed using the Biomark HD instrument. For control purposes, synthetic plasmids (pUC57) containing sequences of all target genes were designed using the Geneious Prime platform (www.geneious.com/prime). The plasmids were cloned into JM109 competent *E. coli*. Extracted plasmids were subjected to the 96.96 Biomark array as described above. Ct values and melting temperature (Tm) were extracted using Biomark Real-Time PCR analysis software. A sample was considered positive to a gene if its Ct value was ≤20 and Tm was in range of average Tm (of all samples with Ct ≤20) ±1°C.^24^ A calibration curve function was built for each of the 94 ARGs by performing serial dilutions of ARG DNA containing known gene copy numbers (Table S2). The number of ARG copies in each sample DNA suspension was extrapolated from the Ct values to the obtained function.

### Data analysis

AMU was expressed in number of ADD in kg (ADD_kg_) per 1000 kg chicken-days, as described previously,^22^ and was summarized by flock by week. The Wilcoxon signed rank test was used to compare AMU between the two periods defined by the sampling schedule: (i) restocking to mid-production (early period); and (ii) mid- to end-production (late period).

ARG negative-testing samples were assigned the ‘0’ value. The estimated number of ARG copies was expressed in relation to: (i) number of copies of 16S rRNA (ARGs/16S rRNA); and (ii) 1 ng of DNA (ARGs/ngDNA). The obtained ARGs/ngDNA values were aggregated by antimicrobial class. The frequency of detection of ARGs between different sampling points (day-old, mid- and end-production) were compared using McNemar’s test. The obtained ARGs/16S rRNA and ARGs/ngDNA values between sampling points were compared using Wilcoxon signed rank test.

Linear mixed-effects models were built to investigate the impact of AMU on: (i) the observed number of copies of 16S rRNA per ngDNA (log_10_); (ii) ARGs/16S rRNA; and (iii) ARGs/ngDNA (log_10_). ‘Farm’ was specified as a random effect and total ADD_kg_ per 1000 chicken-days in the early and late periods for each flock included as separate variables. We allowed the effect of AMU in the early period on gene content to differ between periods by including an interaction term between AMU in early and production periods. For the early period, AMU in the late period was not used by setting its value at zero. The values of 16S rRNA/ngDNA, ARGs/ngDNA and ARGs/16S rRNA in day-old samples were included as covariates in their respective models.

To investigate the impact of the intervention on ARG content, linear mixed-effects models were also built, with ARGs/ngDNA (log_10_) and ARGs/16S rRNA as outcomes, ‘farm’ specified as random effect and the corresponding values for ARGs/16S rRNA and ARGs/ngDNA in day-old samples were included as covariates. All analyses were done using the statistical software R version 3.6.3,^25^ and nlme^26^ and GLMMadaptive packages.^27^

## Results

### Study flocks

Forty-seven baseline and 36 intervention (total 83) flocks raised in 25 farms were investigated (Figure S1). All farms had at least one baseline and one intervention flock investigated. During the baseline phase, 12 (48%) farms raised one flock, 7 (28%) had two flocks, 3 (12%) had three flocks and 3 (12%) had four flocks. During the intervention phase, 17 (68%) farms raised one, 5 (20%) two flocks and 3 (12%) three flocks. The median number of chickens restocked per flock was 306 (IQR 159–520). The median duration of the flock cycle was 18 (IQR 16–20) weeks. The median age of chickens at the second sampling point (mid-production) was 8 (IQR 7–8) weeks, and at the third sampling (end-production) was 16 (IQR 14–17) weeks.

### AMU

A total of 70 (84.3%) and 40 (48.2%) flocks were administered antimicrobials in the early (between day-old and mid-production) and late period (between mid- and end-production), respectively. Ten flocks received no antimicrobials over the whole production cycle. Weekly estimates of AMU are displayed in Figure S2. Flocks were given a mean of 736.7 [standard error (SE) 83.0] ADD_kg_ per 1000 chicken-days in the early period, compared with 52.1 (SE 9.9) ADD_kg_ per 1000 chicken-days in the late period (*P* < 0.001). A total of 29 antimicrobials (belonging to 12 different classes) were administered (Table 1). AMU was reduced from a mean of 444.1 (SE 54.2) ADD_kg_ in the baseline phase to 323.0 (SE 69.2) ADD_kg_ per 1000 chicken-days (i.e. a modelled adjusted reduction of 74.2%) (*P* = 0.002).

**Table 1.** Antimicrobials administered to study flocks (*N* = 83)

Antimicrobial class (antimicrobials)	Early period		Late period		Baseline		Intervention	
	No. of flocks (%) (*n* = 83)	ADD_kg_ per 1000 chicken-days	No. of flocks (%) (*n* = 83)	No. of ADD_kg_ per 1000 chicken-days	No. of flocks (%) (*n* = 47)	No. of ADD_kg_ per 1000 chicken-days	No. of flocks (%) (*n* = 36)	No. of ADD_kg_ per 1000 chicken-days
Macrolides^a^ (erythromycin, tylosin, tilmicosin, spiramycin, josamycin)	22 (26.5)	50.7 (0–0–9.5)	19 (22.9)	8.3 (0–0–0)	22 (46.8)	32.3 (0–0–41.5)	10 (27.8)	22.1 (0–0–2.3)
Polymyxins^a^ (colistin)	59 (71.1)	254.2 (0–112.3–385.0)	17 (20.5)	6.0 (0–0–0)	42 (89.4)	153.0 (21.8–77.8–225.5)	20 (55.6)	99.3 (0–13.4–114.8)
Quinolones^a^ (enrofloxacin, flumequine, norfloxacin)	17 (20.5)	37.2 (0–0–0)	10 (12.0)	10 (0–0–0)	16 (34.0)	27.5 (0–0–15.3)	7 (19.4)	19.9 (0–0–0)
Aminoglycosides^b^ (gentamicin, neomycin, streptomycin, spectinomycin)	33 (39.7)	38.6 (0–0–24.6)	14 (16.9)	4.3 (0–0–0)	30 (63.8)	22.6 (0–7.7–24.8)	9 (25.0)	17.8 (0–0–0.6)
β-Lactams^b^ (ampicillin, amoxicillin)	19 (22.9)	16.2 (0–0–0)	14 (16.9)	4.6 (0–0–0)	19 (40.4)	11.6 (0–0–16.6)	8 (22.2)	7.8 (0–0–0)
Phenicols^c^ (florfenicol, thiamphenicol)	10 (12.0)	18.9 (0–0–0)	5 (6.0)	1.4 (0–0–0)	10 (21.3)	5.6 (0–0–0)	4 (11.1)	18.9 (0–0–0)
First-generation cephalosporins^c^ (cefadroxil)	0 (0)	0	1 (1.2)	0.5 (0–0–0)	1 (2.1)	0.5 (0–0–0)	0 (0)	0
Folate pathway inhibitors^c^ (trimethoprim)	13 (15.7)	18.1 (0–0–0)	7 (8.4)	2.3 (0–0–0)	12 (25.5)	12.6 (0–0–0.7)	5 (13.9)	6.0 (0–0–0)
Lincosamides^c^ (lincomycin)	2 (2.4)	0.3 (0–0–0)	5 (6.0)	1.7 (0–0–0)	6 (12.8)	1.9 (0–0–0)	1 (1.8)	0.1 (0–0–0)
Sulphonamides^c^ (sulfadimidine, sulfathiazole, sulfamethoxazole, sulfadimethoxine, sulfadiazine)	18 (21.7)	22.3 (0–0–0)	8 (9.6)	1.0 (0–0–0)	16 (34.0)	13.9 (0–0–2.2)	7 (19.1)	9.4 (0–0–0)
Tetracyclines^c^ (doxycycline, oxytetracycline, tetracycline)	60 (72.3)	278.9 (0–158.9–439.8)	23 (27.7)	10.6 (0–0–8.4)	41 (87.2)	161.9 (28.6–80.7–259.1)	24 (66.7)	121.6 (0–60.1–145.6)
Methenamine	1 (1.2)	0.5 (0–0–0)	1 (1.2)	0.2 (0–0–0)	1 (2.1)	0.7 (0–0–0)	0 (0)	0
Any antimicrobial	70 (84.3)	736.7 (184.3–529.1–1172.8)	40 (48.2)	52.1 (0–0–71.5)	46 (97.9)	444.1 (147.9- 303.8–650.7)	27 (75.0)	323.0 (17.8–175.4–386.6)

Weekly average ADD_kg_ per 1000 chicken-days were expressed as mean as well as 1st quartile - median - 3rd quartile (in brackets).

a Antimicrobial classes categorized according to WHO (2018) as ‘highest priority critically important’.

b High priority.

c Critically important and highly important.

### Assessment of DNA and bacterial biomass over time

Assuming that each sample contained 25 g of faecal material, and accounting for subsequent dilutions of the sample, the DNA concentration was highest in end-production faecal samples [average 72.7 (SE 7.2) ng/mg of matrix], followed by mid-production [56.2 (SE 7.5)] and day-old [33.1 (SE 6.2) ng/mg]. The number of 16S rRNA copies per ngDNA (log_10_) increased from day-old samples [mean 5.97 (SE 0.13)] to mid-production samples [mean 6.72 (SE 0.06), *P* = 0.004], subsequently decreasing at end-production [mean 6.52 (SE 0.07), *P* = 0.188].

There was good correlation between ARGs/ngDNA and 16S rRNA/ngDNA (Spearman’s correlation, *ρ* = 0.95, *P* < 0.001).

### Differences in DNA and bacterial biomass between baseline and intervention

Intervention-phase samples contained (not significant) higher amounts of DNA [mean 57.0 (SE 6.5 ng/mg)] than baseline-phase samples [51.7 (SE 5.4) ng/mg, *P* = 0.442]. The mean 16S rRNA/ngDNA (log_10_) was higher in intervention-phase [6.52 (SE 0.08)] compared with baseline-phase samples [6.31 (SE 0.08), *P* = 0.087]. Specifically, 16S rRNA/ngDNA (log_10_) in day-old samples was higher in intervention [6.21 (SE 0.18)] than in baseline flocks [5.78 (SE 0.18), *P* = 0.081].

### Changes in ARGs over the flock cycle

**Figure 1.**
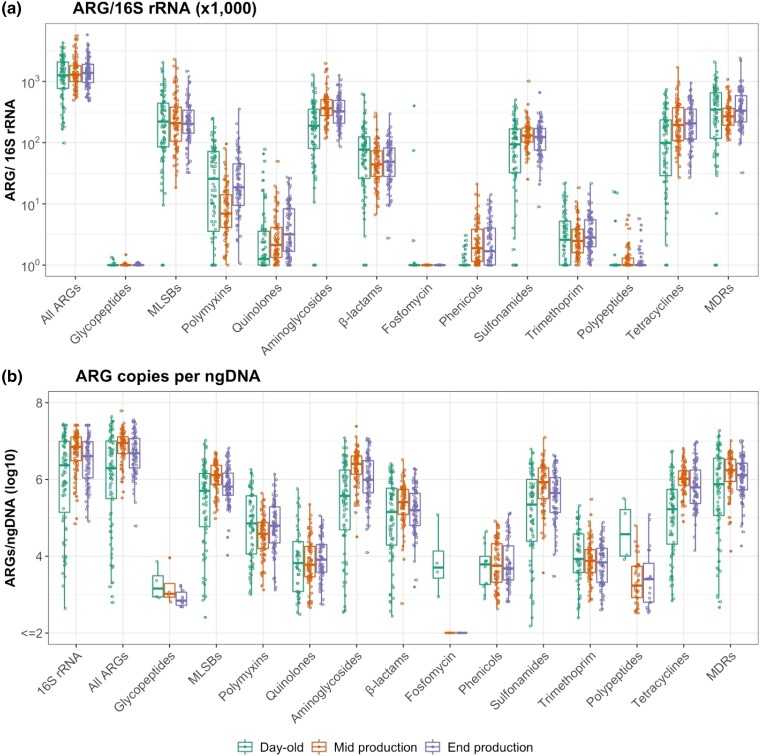
(a) ARG/16S rRNA and (b) ARGs/ngDNA (log_10_) in chicken faecal samples. MLS_B_, macrolide/lincosamide/streptogramin B. The boxes indicate the median and IQR values.

There was a slight increase in overall ARGs/16S rRNA from day-old (average) [1.47 (SE 0.10)] to mid-production samples [1.61 (SE 0.16) *P* = 0.638], and a further reduction in end-production [1.60 (SE 0.1), *P* = 0.645] (Figure 1 and Figure S3). Overall ARGs/ngDNA increased from mean (log_10_) 6.02 (SE 0.14) in day-olds to 6.86 (SE 0.05) (log_10_) in mid-production (*P* = 0.001), subsequently decreasing to 6.67 (SE 0.06) end-production samples (*P* = 0.074) (Figure 1 and Table S3).

**Figure 2.**
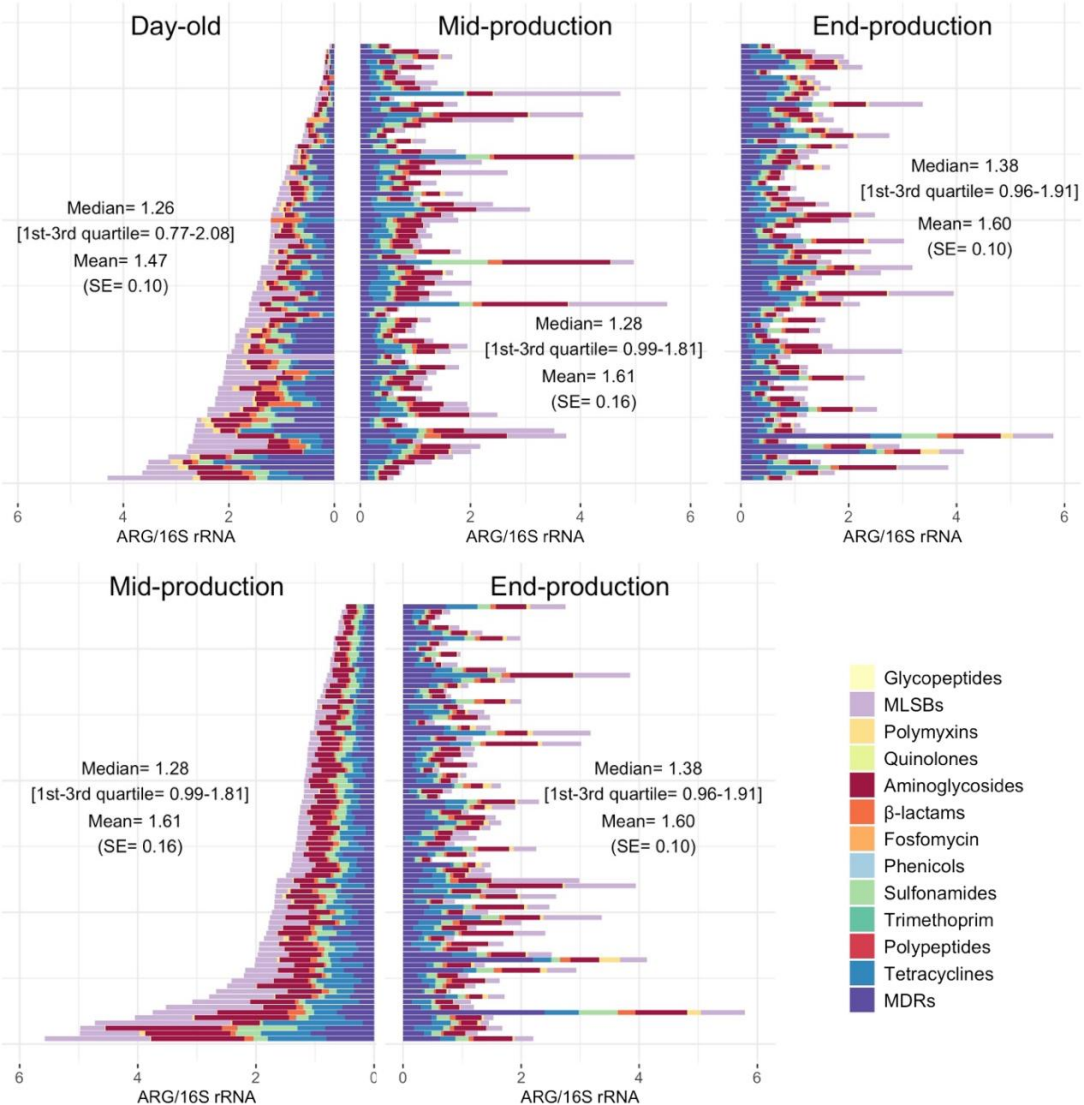
Sum of ARGs/16S rRNA by class by flock over the production cycle. Each horizontal bar represents one flock. Results for each flock over subsequent sampling points are aligned.

ARGs/16S rRNA increased in mid-production for aminoglycosides (*P* < 0.001), phenicols (<0.001), sulphonamides (*P* = 0.002) and tetracyclines (*P* = 0.006). In contrast, reductions were observed for polymyxins (*P* < 0.001), β-lactams (*P* = 0.003) and multi-drug resistance (MDR) (*P* = 0.041). At end of the production cycle, ARGs/16S rRNA decreased for aminoglycoside resistance (*P* = 0.04) but increased for polymyxin (*P* < 0.001) and quinolone resistance (*P* = 0.033). Figure 2 shows changes of ARGs by class in relation to 16S rRNA over the production cycle.

Expressed in relation to ngDNA, ARG quantities increased for resistance against MLS_B_s (*P* = 0.002), quinolones (*P* = 0.015), aminoglycosides (*P* < 0.001), phenicols (*P* < 0.001), sulphonamides (*P* < 0.001), tetracyclines (*P* < 0.001) and polypeptides (*P* < 0.001) between day-olds and mid-production. Reductions in ARGs/ngDNA in end-production samples were seen for MLS_B_s (*P* = 0.023), aminoglycosides (*P* = 0.010), β-lactams (*P* = 0.048) and sulphonamides (*P* = 0.009). Changes in individual ARGs (both prevalence and in relation to 16S rRNA and DNA) are shown in Table S1 and Figure S4.

There was a weak correlation of overall ARGs/ngDNA between day-old and mid-production (Spearman’s correlation, *ρ* = 0.199, *P* = 0.07) and between mid- and end-production samples (Spearman’s correlation, *ρ* = −0.09, *P* = 0.40) from the same flocks (data not shown).

### Association between AMU and ARGs

AMU led to quantitative increases of ARGs/16S rRNA by 4.1% and 16.3% in the early and late periods, respectively (both *P* > 0.05). AMU during the late period resulted in a 53% reduction in 16S rRNA/ngDNA, as well as a reduction of 30.8% in ARGs/16S rRNA (compared with flocks that received no antimicrobials) (Table 2).

**Table 2.** Predicted 16S rRNA/ngDNA, ARGs/ngDNA and ARGs/16S rRNA mean values (95% confidence intervals) in flocks resulting from AMU (391.6 ADDs per 1000 chicken-days) in early and late-production samples as well as the corresponding values in day-old samples

Models	Predicted outcome	Degree of change (%)	*P* value
Outcome 1: 16S rRNA/ngDNA (log_10_)			
Mid-production			
No AMU	6.67 (6.47–6.87)	—	—
AMU in early period	6.69 (6.62–6.76)	+4.7	0.639
1 unit of 16S rRNA/ngDNA in day-olds^a^	6.69 (6.62–6.76)	+4.7	0.537
End production			
No AMU	6.52 (6.32–6.73)		
AMU in early period	6.53 (6.48–6.60)	+2.3	0.728
AMU in late period	6.17 (5.59–6.75)	−53.3	0.231
1 unit of 16S rRNA/ngDNA in day-olds^a^	6.54 (6.47–6.61)	+4.7	0.537
Outcome 2: ARGs/16S rRNA			
Mid-production			
No AMU	1.47 (1.04–1.90)	—	—
AMU in early period	1.53 (1.41–1.65)	+4.1	0.353
1 unit of ARGs/16S rRNA in day-olds^b^	1.50 (1.24–1.75)	+2.0	0.830
End-production			
No AMU	1.35 (0.92–1.78)	—	—
AMU in early period	1.45 (1.33–1.55)	+7.4	0.113
AMU in late period	1.55 (0.59–2.54)	+16.3	0.664
1 unit of ARGs/16S rRNA in day-olds^b^	1.38 (1.12–1.63)	+2.2	0.830
Outcome 3: ARGs/ngDNA (log_10_)			
Mid-production			
No AMU	6.79 (6.63–6.95)	—	—
AMU in early period	6.81 (6.76–6.87)	+4.7	0.452
1 unit of ARGs/ngDNA in day-olds^c^	6.82 (6.77–6.87)	+7.1	0.232
End production			
No AMU	6.62 (6.45–6.78)		
AMU in early period	6.64 (6.58–6.70)	+4.7	0.465
AMU in late period	6.46 (6.0–6.92)	−30.8	0.493
1 unit of ARGs/ngDNA in day-olds^c^	6.65 (6.60–6.70)	+7.1	0.232

a 1 unit = 6.83 log_10_.

b 1 unit = 1.47.

c 1 unit = 6.77 log_10_.

### Impact of the intervention on ARGs

**Figure 3.**
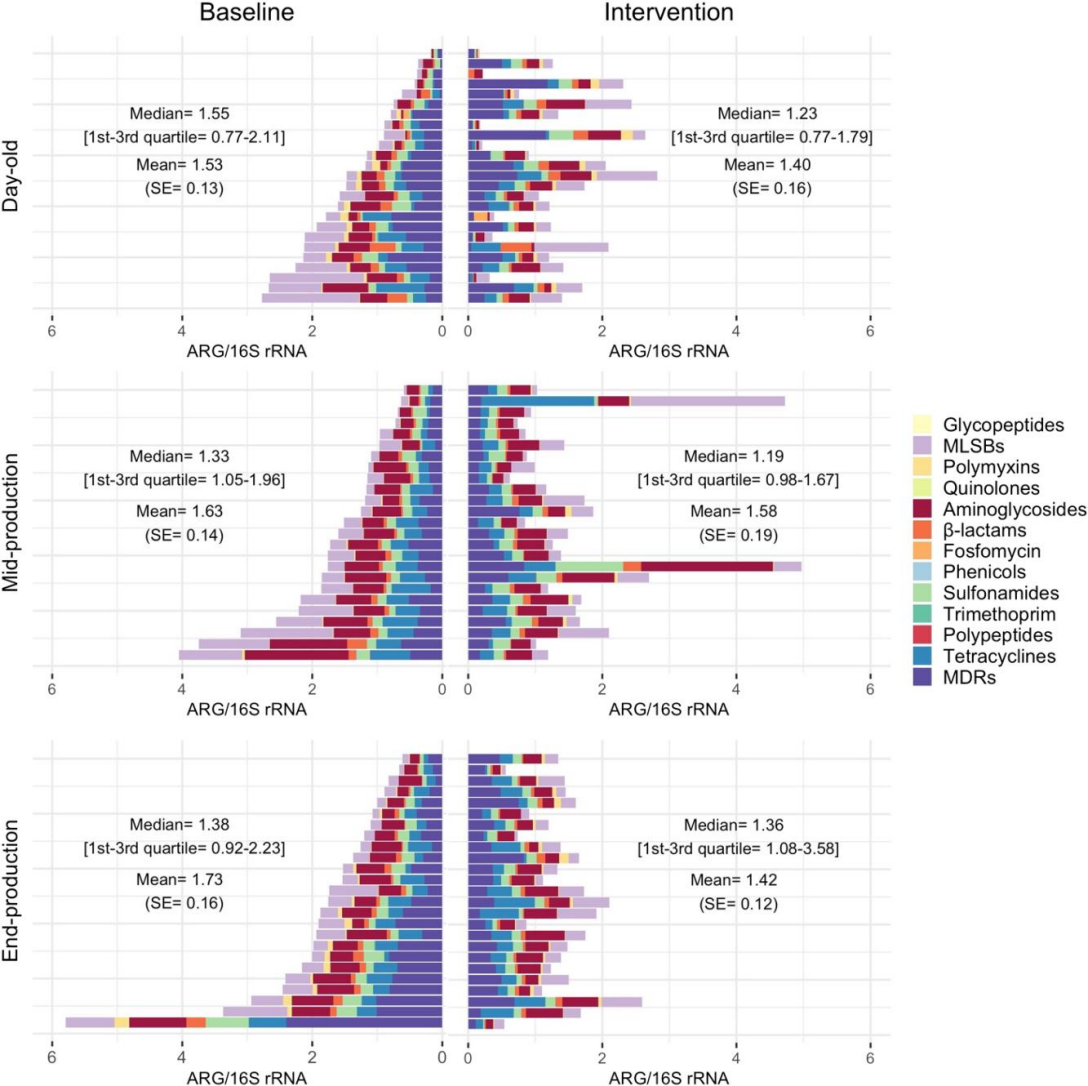
Sum of ARGs/16S rRNA by class by farm during the baseline and intervention phases. For farms raising more than one flock in each phase, the corresponding flock values were averaged. Each horizontal bar represents one farm. Results for each farm over the baseline and intervention phases are aligned.

The data on ARGs/ngDNA and ARGs/16S rRNA from baseline and intervention flocks by class are shown in Figure 3 and Figure S5, respectively.

In mid-production samples, ARGs/16S rRNA from intervention flocks (compared with baseline) was reduced for 34 (42.5%), increased for 42 (52.5%) and did not change (or was not detected) for 4 (5%) of the ARGs investigated. In end-production, ARGs/16S rRNA was reduced for 60 (75%), increased for 16 (29%) and did not change (or was not detected) for 4 (5%) ARGs. In mid-production, ARGs/ngDNA from intervention flocks (compared with baseline) was reduced for 40 (50%) ARGs, increased for 34 (42.5%) genes, and did not change (or was not detected) for 6 (7.5%) genes. In end-production, ARGs/ngDNA was reduced for 27 (33.7%), increased for 47 (58.8%) and did not change (or was not detected) for 6 ARGs (7.5%) (Table S4).

The model resulted in an overall 10.7% reduction in ARGs/16S rRNA (*P* = 0.243). By antimicrobial class, the reductions ranged between 0% and 17.6% (all *P* > 0.05). In contrast, the models predicted a 14.8% increase of ARGs/ngDNA as a result of the intervention (*P* = 0.464) (Table 3).

**Table 3.** Predicted ARG/16S rRNA and ARG/ngDNA (log_10_) means (95% confidence intervals) resulting from the intervention for all ARGs and per antimicrobial class

Antimicrobial class	ARG/16S rRNA				ARG/ngDNA (log_10_)			
	Baseline	Mid- and end-production	Degree of change (%)	*P* value	Baseline	Mid- and end-production	Degree of change (%)	*P* value
For all ARGs	1.68	1.50 (1.20–1.80)	−10.7	0.243	6.78	6.84 (6.68–6.99)	+14.8	0.464
MLS_B_	0.34	0.28 (0.18–0.39)	−17.6	0.291	5.95	5.95 (5.81–6.10)	+0.8	0.961
Polymyxins	0.02	0.02 (0–0.03)	0	0.588	4.62	4.62 (4.40–4.84)	+0.8	0.975
Quinolones	0.04	0.04 (0.02–0.06)	0	0.865	3.57	3.55 (3.17–3.93)	+4.7	0.916
Aminoglycosides	0.43	0.37 (0.29–0.45)	−13.9	0.166	6.14	6.18 (6.01–6.35)	+9.6	0.666
β-Lactams	0.07	0.06 (0.04–0.07)	−14.3	0.078	5.27	5.27 (5.09–5.46)	+1	0.961
Phenicols	0.02	0.02 (0.01–0.03)	0	0.389	3.09	2.90 (2.39–3.40)	−54.9	0.447
Sulphonamides	0.14	0.14 (0.12–0.18)	0	0.524	5.66	5.77 (5.58–5.99)	+28.8	0.254
Trimethoprim	0.03	0.03 (0.02–0.04)	0	0.490	3.74	3.72 (3.46–3.98)	−4.7	0.899
Tetracyclines	0.27	0.25 (0.19–0.32)	−7.4	0.645	5.90	5.92 (5.76–6.07)	+4.7	0.844
MDR	0.40	0.35 (0.26–0.45)	−12.5	0.324	6.07	6.15 (5.99–6.31)	+20.2	0.318

MLS_B_, macrolide/lincosamide/streptogramin B; MDR, multi-drug resistance.

## Discussion

Our findings confirmed a wide diversity of ARGs in the tested samples, reflecting the complex microbiota and metagenomics in the chicken’s gut,^28^ as well as its evolving dynamics over the flock production cycle.^29–31^ Specifically, *E. coli*, *Klebsiella*, *Enterococcus*, *Staphylococcus* and *Lactobacillus* have been identified as important ARG reservoirs.^32^

The prevalence of ARGs clearly increased between day-old and mid-production (∼2–3 months); however, these changes were much more marked if expressed in relation to DNA.

Also the models investigating the impact of AMU on ARGs gave contradictory (albeit non-significant) results depending on the metric of choice. Data on 16S rRNA related to DNA suggest that samples in mid- production had the greatest bacterial biomass. This may explain why changes of ARG were much more evident when expressed in relation to DNA than to 16S rRNA. Indeed, we found a high correlation between 16S rRNA and ARG content, indicating that bacterial biomass in samples (any genus) is a good predictor of ARGs.

The reasons for the increased changes in the DNA content of samples over time are unknown. Also, the differences in DNA between intervention and baseline samples in DNA content are intriguing, since the methodology was kept constant over the study period. In order to calculate ngDNA per mg of faeces, we assumed all samples contained 25 g (from previous study, data not shown). One possible explanation is that this is a reflection of varying amounts of matrix in the sample.

Although our intervention resulted in measurable overall reductions in AMU,^22^ we found in intervention flocks a modest (albeit not statistically significant) reduction in ARGs expressed in relation to 16S rRNA (proxy of bacterial content), but an overall (also non-significant) increase in ARGs in relation to DNA. In the intervention flocks, 16S rRNA was increased, probably reflecting less disruption of bacterial populations due to lesser AMU levels, and may partly explain the difference between the two metrics.

Previous studies at colony level (*E. coli*) have reported increases in the prevalence of phenotypic resistance to most antimicrobials in Vietnamese meat chicken flocks between Day 0 and Days 25–48.^15^ In contrast, *E. coli* from layer flocks in Spain displayed the highest prevalence of ARGs at day-old, decreasing thereafter.^16^ The changes in prevalence of colonization over time suggest that the slaughter age of birds may also condition the risk of potential transfer of ARG-harbouring bacteria to in-contact slaughterers and consumers.^33^ Unlike broilers, chickens in our study were slow-growing native breeds that were slaughtered at 4–5 months with ARG abundance being highest in mid-production (∼2.5 months). Therefore, we suggest that earlier slaughtering of these birds may potentially increase the risks to in-contact humans and consumers.

We observed lower DNA values, but higher 16S rRNA/ngDNA and ARGs/ngDNA values in day-old chickens purchased during the intervention phase compared with the baseline phase (data not shown). We speculate that this may be a reflection of changes in AMR colonization (possibly due to AMU) in parent flocks later in time (since our study was conducted over a 3 year period).

Published AMU-reducing interventions on food-producing animals result in variable reductions in prevalence of phenotypic AMR depending on individual antimicrobials.^34^ For example a 57% reduction of AMU in Dutch broilers from 2009 to 2014 resulted in relative decreases in the prevalence of resistance from 8% to 31%.^35^ It is also noteworthy that, in contrast with other studies, ours was conducted in small-scale farming systems typical of developing country settings. Most, if not all, farms in our study had very poor hygiene and biosecurity measures, and water was often sourced from a river or canal, which is common practice in the Mekong Delta region.

We detected many ARGs encoding resistance against CIAs [i.e. colistin (*arnA*, *mcr-1*), MLS_B_ (*erm* genes)] and ESBLs (*bla*_CTX-M_ and *bla*_SHV_) at extremely high prevalence (>40%) in day-old samples. ARGs carried in the flora of day-old chicks is likely to reflect colonization in the hatchery environment or parent flock.^36^ Some studies have shown that hatcheries are important AMR sources to broiler farms.^16,37^ A study in Korea showed that the use of ceftiofur in hatcheries resulted in colonization with flora resistance to third-generation cephalosporins.^37^ In small-scale production systems in our study, day-old chicks were procured through informal channels. Therefore, it is also possible that chicks may become colonized during the transport and distribution stages. We could not, however, demonstrate a substantial impact of day-old ARGs on subsequent ARG levels in mid- and end-production in study flocks.

We found that 48.2% (120/249) of samples contained the *mcr-1* gene, which is consistent with a previous study in Vietnam.^21^ Even though the prevalence of the *mcr-1* gene slightly increased from day-old (54%) to mid-production (60%) samples, the number of copies of this gene in positive samples decreased considerably (from 3.76 to 3.29 log_10_). A similar result was observed for the *arnA* gene, which was by far the most abundant of all polymyxin resistance-encoding genes. We also identified the *mcr*-3 gene in 1/249 samples. This gene has been previously detected in meat products (other than chicken meat) in Vietnam.^38,39^

We found weak evidence of an impact of concurrent AMU on ARGs. A study of chicken flocks in nine European countries reported associations between the use of β-lactams, tetracyclines, macrolides and lincosamides, trimethoprim and aminoglycosides, and abundance of their corresponding ARGs, but also high frequency of ARGs in flocks that had not been treated with antimicrobials.^19^ Indeed, many AMR mechanisms result in negligible fitness costs or are even cost-free.^40,41^ Furthermore, the association between AMU and AMR may be confounded by longitudinal changes in the birds’ gut microbiota,^30^ as well as by cross-resistance selection effects.^40^ In addition to the weak evidence from modelling results, we found little correlation between ARG content in flocks over time, suggesting high turnover of ARGs in flocks over time. This strongly suggests that, in addition to AMU practices, flocks may acquire ARGs through contaminated water/feed, the environment, from residual contamination of previous flocks housed in the same buildings or from other animal sources.^42^ Because of this, in addition to promoting responsible AMU, stepping up biosecurity, cleaning and disinfection and biocontainment should be a priority to mitigate generation and transmission of AMR in small-scale poultry flocks.

## Supplementary Material

dlad090_Supplementary_Data

## Supplementary data

Figures S1 to S5 and Tables S1 to S4 are available as Supplementary data at *JAC-AMR* Online.
