# Supplementary material for: Impact of antimicrobial use on abundance of antimicrobial resistance genes in chicken flocks in Vietnam
Source: JAC Antimicrob Resist. 2023 Jul 22;5(4):dlad090. doi: 10.1093/jacamr/dlad090 (PMC10362913; doi:10.1093/jacamr/dlad090)
Supplement: dlad090_Supplementary_Data [file dlad090_supplementary_data.zip › ResistantGene_Suppl_Figures_JAC_AMR_r2_F.docx]

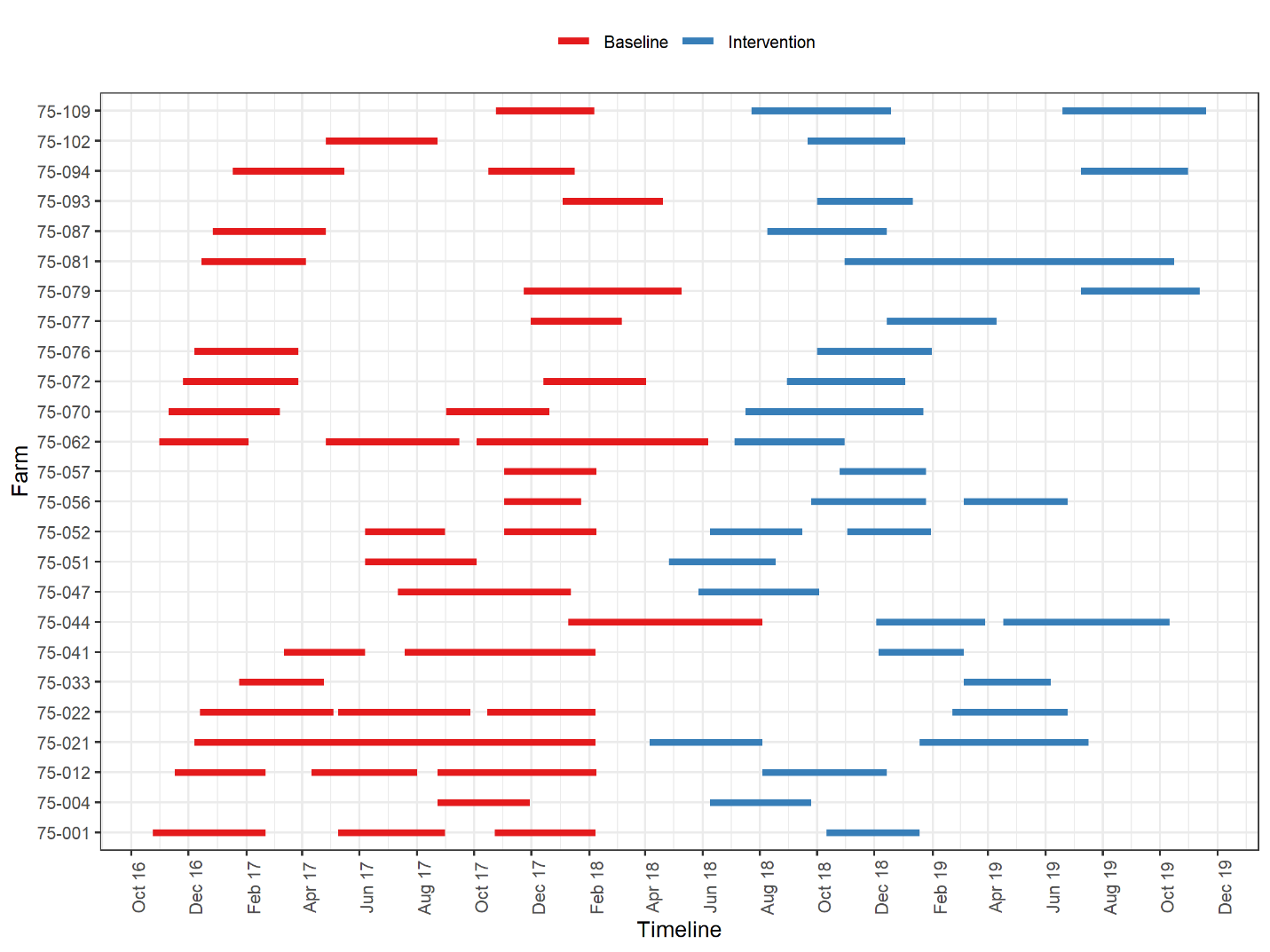


**Figure S1**. Timeframe of recruitment of flocks.


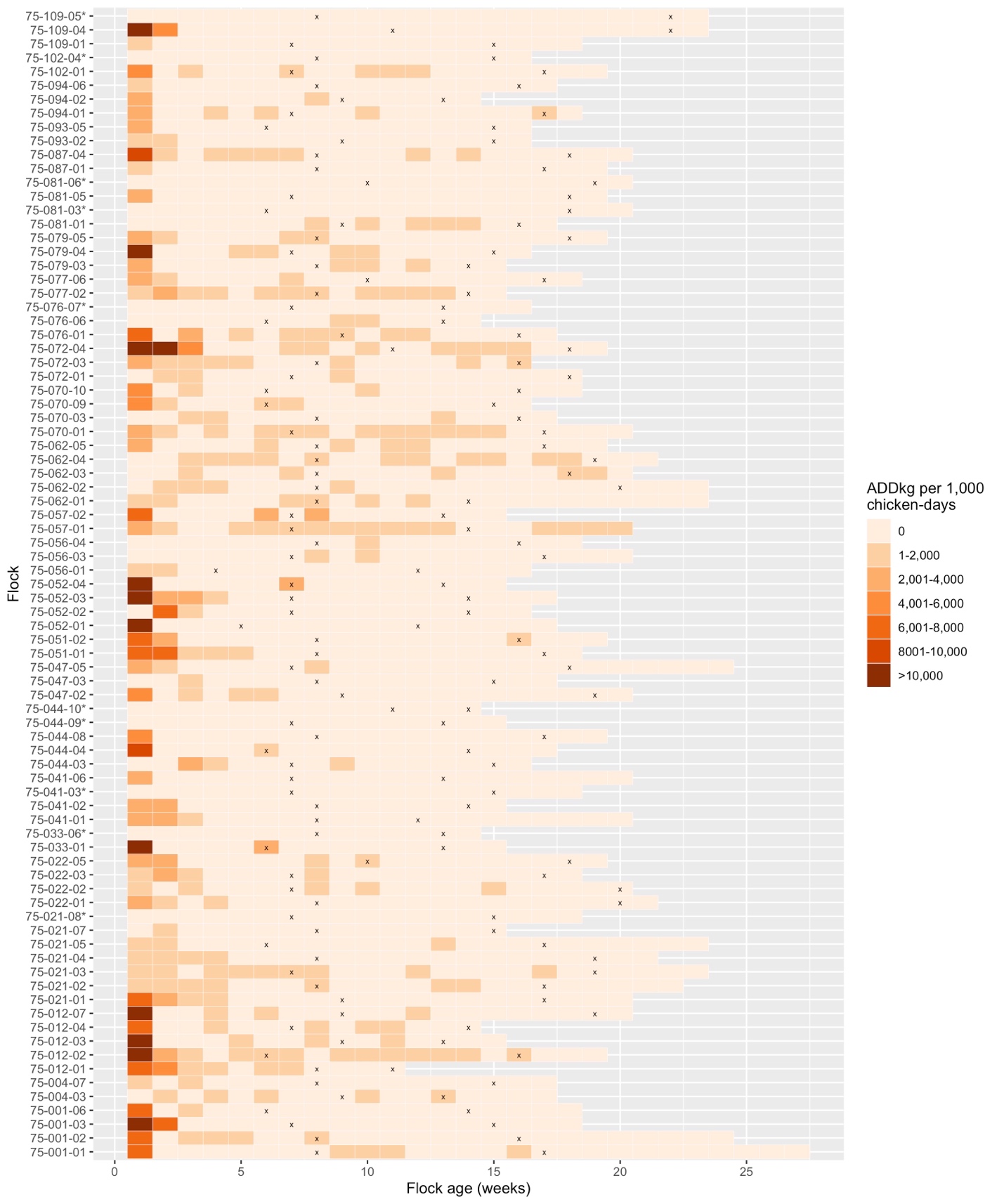


**Figure S2**. Estimates of AMU (ADD_kg_ per 1,000 chicken-days) per week for 83 study flocks. Each horizontal line represents one flock. The crosses indicate the week of sampling. An asterisk (*) next to the flock ID name indicates no treatment.


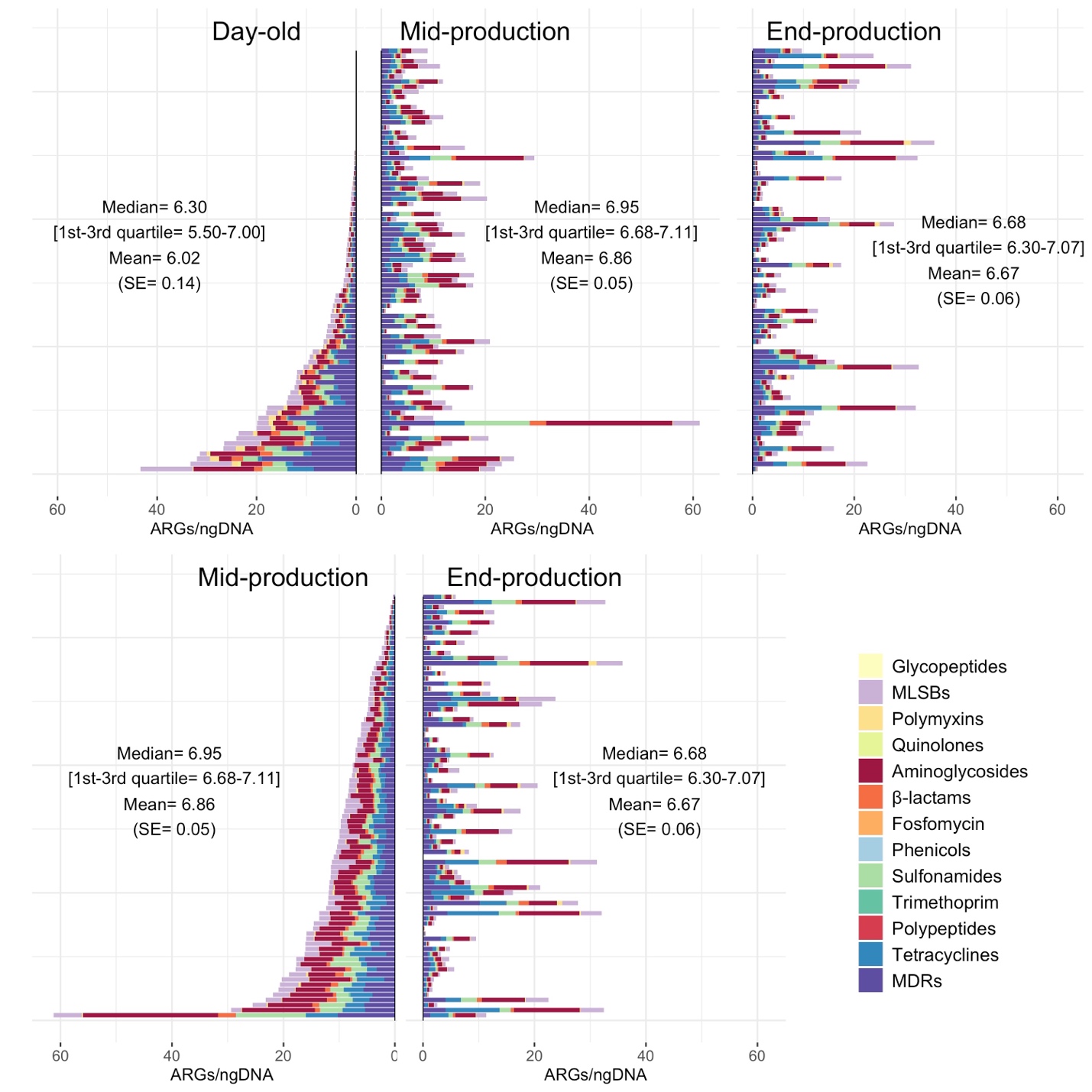


**Figure S3.** ARGs/ngDNA by class by flock over the production cycle. For better visualization, ARGs/ngDNA were re-scaled by dividing to 10^6^.


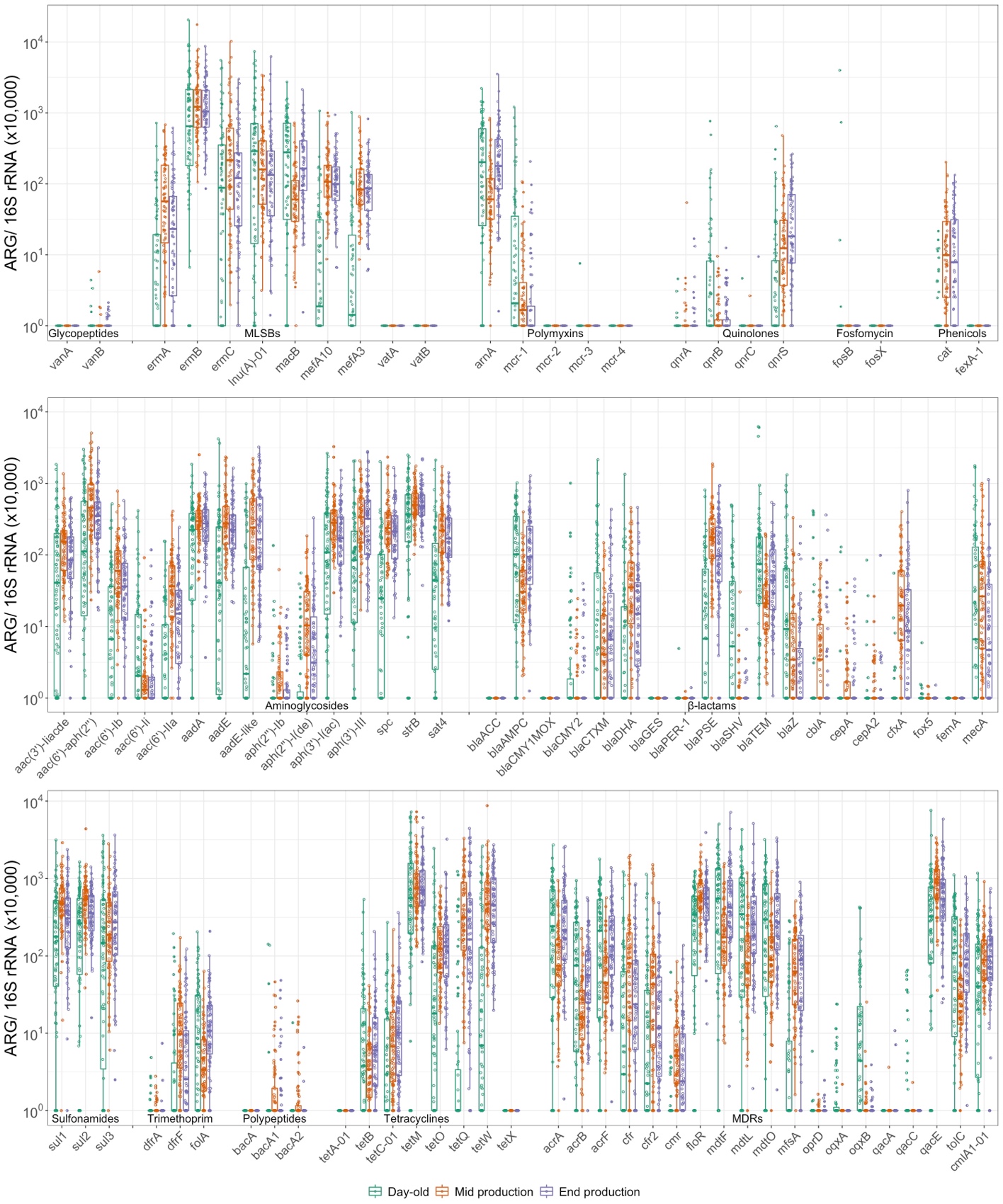


**Figure S4.** ARGs/16S rRNA in chicken faecal samples over the flock production cycle. MLSB= Macrolide-Lincosamide-Streptogramin B, MDR= multidrug resistance.

**
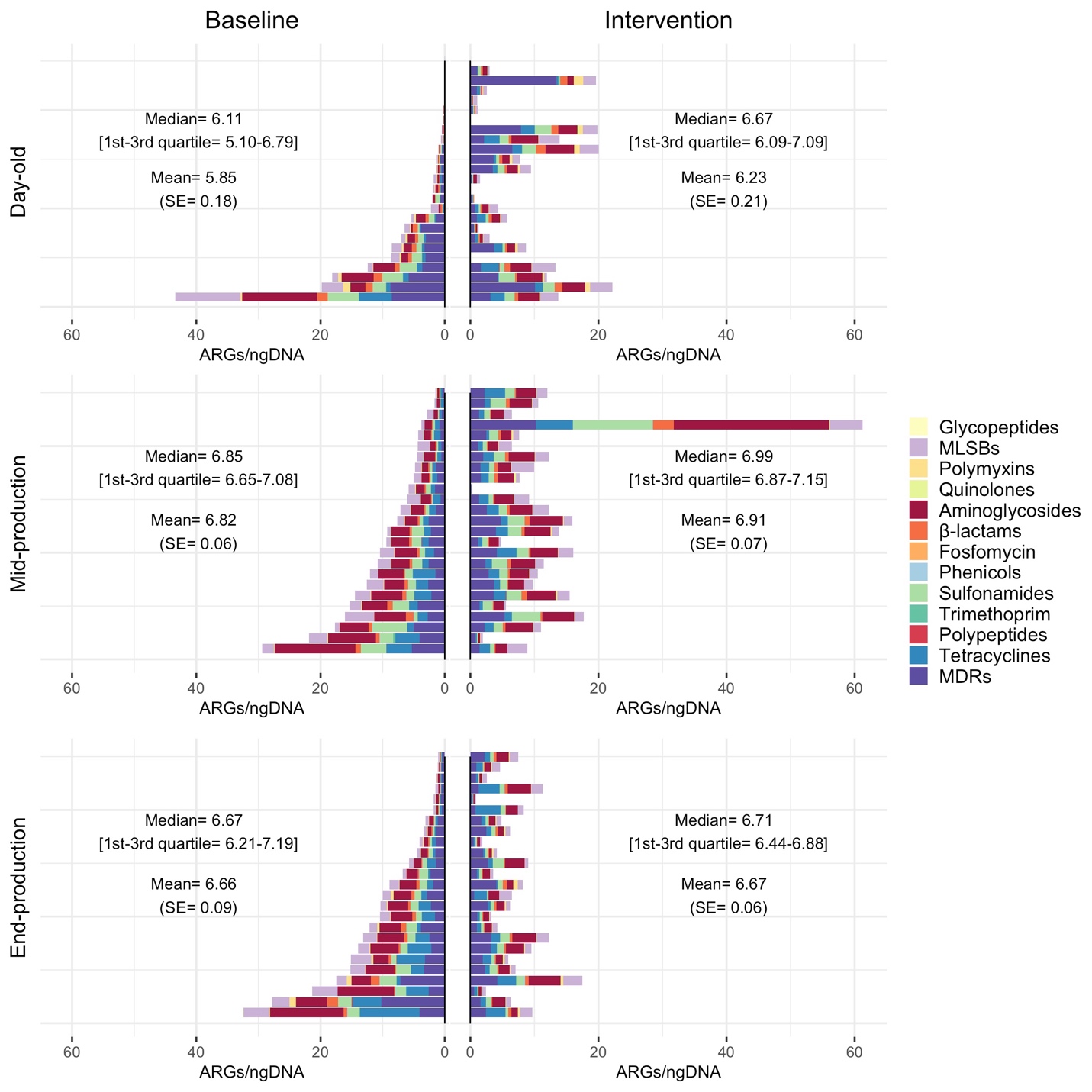
**

**Figure S5.** Average of ARGs/ngDNA by class by flock for the baseline and intervention phases. For farms raising more than one flock in each phase, ARG abundance was averaged. For easier visualization, ARGs/ngDNA were re-scaled by dividing by 10^6^.
